# Exogenous Vitamins K Exert Anti-Inflammatory Effects Dissociated from Their Role as Substrates for Synthesis of Endogenous MK-4 in Murine Macrophages Cell Line

**DOI:** 10.3390/cells10071571

**Published:** 2021-06-22

**Authors:** Anna Kieronska-Rudek, Agnieszka Kij, Patrycja Kaczara, Anna Tworzydlo, Marek Napiorkowski, Katarzyna Sidoryk, Stefan Chlopicki

**Affiliations:** 1 Jagiellonian Centre for Experimental Therapeutics (JCET), Jagiellonian University, Bobrzynskiego 14, 30-348 Krakow, Poland; anna.kieronska@jcet.eu (A.K.-R.); agnieszka.kij@jcet.eu (A.K.); patrycja.kaczara@jcet.eu (P.K.); anna.tworzydlo@jcet.eu (A.T.); 2 Department of Pharmacology, Medical College, Jagiellonian University, Grzegorzecka 16, 31-531 Krakow, Poland; 3 Chemistry Department, Pharmaceutical Research Institute, Rydygiera 8, 01-793 Warszawa, Poland; mk.napiorkowski@gmail.com (M.N.); k.sidoryk@ifarm.eu (K.S.)

**Keywords:** macrophages, inflammation, vitamins K, endogenous MK-4, statin

## Abstract

Vitamins K exert a range of activities that extend far beyond coagulation and include anti-inflammatory effects, but the mechanisms involved in anti-inflammatory action remain unclear. In the present study, we showed that various forms of exogenous vitamins—K_1_, K_3_, K_2_ (MK-4, MK-5, MK-6 and MK-7)—regulated a wide scope of inflammatory pathways in murine macrophages in vitro, including NOS-2, COX-2, cytokines and MMPs. Moreover, we demonstrated for the first time that macrophages are able to synthesise endogenous MK-4 on their own. Vitamins with shorter isoprenoid chains—K_1_, K_3_ and MK-5—exhibited stronger anti-inflammatory potential than vitamins with longer isoprenoid chains (MK-6 and MK-7) and simultaneously were preferably used as a substrate for MK-4 endogenous production. Most interesting, atorvastatin pretreatment inhibited endogenous MK-4 production but had no impact on the anti-inflammatory activity of vitamins K. In summary, our results demonstrate that macrophages are able to synthesise endogenous MK-4 using exogenous vitamins K, and statin inhibits this process. However, the anti-inflammatory effect of exogenous vitamins K was independent of endogenous MK-4 synthesis.

## 1. Introduction

Vitamins K was discovered in 1929 as a substance essential for blood coagulation and was named the “coagulation vitamin.” Today, it is well known that “vitamins K” encompasses a group of fat-soluble 2-methyl-1,4 naphthoquinone derivatives with isopropyl side chains, including phylloquinone (vitamin K_1_), menaquinones (vitamin K_2_) and menadione (vitamin K_3_) [1]. The mechanism of activity of vitamins K is attributed to the reduced form of vitamins K (KH_2_) that activates blood coagulation factors (prothrombin, factor VII, IX, X) through post-translational modification of vitamins K-dependent proteins (VKDPs) by γ-glutamyl carboxylase (GGCX), which converts glutamate (Glu) residues to γ–carboxyglutamate (Gla). Hence, vitamins K antagonists inhibit coagulation by targeting vitamins K reduction and the warfarin-sensitive enzyme VKOR (vitamins K epoxide reductase) during the “vitamins K cycle” [2]. Vitamins K-dependent γ–carboxylation was described also for anticoagulant proteins (protein C and protein S) and a variety of other VKDPs identified in extrahepatic tissues. At present, there are at least 17 types of VKPDs reported with various functions. For example, osteocalcin (OC) regulates bone metabolism and insulin sensitivity, matrix Gla protein (MGP) is involved in vascular calcification, growth arrest-specific protein-6 (Gas6) is linked to inflammation and platelet aggregation, and periostin is critical to heart remodelling [3]. In fact, evidence has accumulated that vitamins K exerts a range of activities that extend far beyond coagulation. Vitamins K-dependent mechanisms might play a role not only in the regulation of coagulation factor synthesis in the liver but also in a number of other processes and diseases, such as osteoporosis, osteoarthritis, cancer, diabetes, cognition, vascular calcification [4,5,6,7,8] and inflammation [9,10,11,12].

Indeed, the anti-inflammatory activity of vitamin K_1_ was demonstrated in vivo in rats with endotoxemia, as evidenced by a decreased mRNA level of macrophage migration inhibitory factor in vitamin K_1_-treated animals [12]. The anti-inflammatory effect of vitamin K_1_ was also reported in humans, as evidenced by the inverse correlation between plasma phylloquinone/phylloquinone intake and circulating IL-6 [9]. Nevertheless, evidence supporting the anti-inflammatory activity of vitamins K in immune cells is limited, and the mechanisms involved are not entirely clear. The in vitro studies demonstrated that vitamins MK-3, MK-4 and MK-7 decreased cytokine production (IL-1, IL-6 and TNFα) in macrophages and macrophage-like cells [10,13]. Moreover, Cavaco et al. reported on the anti-inflammatory activity of exogenous Gla-rich protein (GRP) that resulted in the reduction of PGE_2_ production and matrix metalloproteinase 13 gene expression in chondrocytes and synoviocytes stimulated with IL-1β [14].

Accordingly, these studies seem to suggest that apart from the canonical mechanism of VKD carboxylation of proteins [8], other non-canonical mechanisms of activity of vitamins K could be involved in the anti-inflammatory effects of vitamins K. Non-canonical mechanisms include regulation of NFκB activity suggested for macrophages [13] or the regulation of mitochondrial function suggested for other types of cells [15,16,17].

The aim of this work was to characterise more comprehensively than in previous studies [10,13,14] the anti-inflammatory effect of a wide range of forms of vitamins: K_1_, K_3_, MK-4, MK-5, MK-6 and MK-7 in murine macrophages RAW 264.7 cell line. We studied the ability of these vitamins to inhibit the induction of nitric oxide synthase 2 (NOS-2) and NO production, induction of cyclooxygenase 2 (COX-2) and eicosanoid production, cytokine production and MMP activation in macrophages stimulated by LPS. In particular, we examined the possibility that the mechanism of anti-inflammatory action of vitamins K in the macrophages RAW 264.7 cell line would be mediated by endogenous vitamin MK-4 derived from exogenous vitamins K analogues. Altogether, our results showed that the anti-inflammatory effects of vitamins K in the murine macrophages cell line, RAW 264.7 cells could not be attributed to regulation of mitochondrial function, and endogenous MK-4. The anti-inflammatory effect of exogenous vitamins K are most likely linked to the inhibition of nuclear translocation of NFκB [12,13] but not to VKD-carboxylation-dependent mechanisms [13,14]. At the same time, we demonstrated that macrophages displayed active MK-4 synthesis from some, but not all, forms of exogenous vitamins K and that statin inhibited endogenous MK-4 synthesis.

## 2. Materials and Methods

### 2.1. Cell Lines and Cell Culture

The murine macrophage cell line—RAW 264.7—was purchased from the European Collection of Authenticated Cell Cultures (ECCAC, Salisbury, England, UK). Cells were cultured in Dulbecco’s Modified Eagle Medium DMEM (Gibco, Paisley, Scotland, UK) and supplemented with 10% fetal bovine serum (Gibco, Paisley, Scotland, UK) at 37 °C in a humidified atmosphere containing 5% CO_2_. In all experiments, the cells between the first and third passage were used.

### 2.2. Synthesis of Various Forms of Vitamins K

The menadione (K_3_) was purchased from Sigma-Aldrich; vitamin K_1_ was bought from Hefei TNJ Chemical Industry Co., Ltd. (Hefei, China).; and MK-4, MK-5, MK-6 and MK-7 were synthesised based on an approach described in patent No. WO/2014/058330. In brief, synthesis consisted of the following key steps: conversion of menadione into a sulfone-activated analogue of vitamin MK-1, the formation of a polyisoprenoid chain of the desired length, joint of both building blocks and completion of the structure by removing sulfones and protecting groups. Vitamin MK-7 was synthesised directly via the method referred to in the patent as “variant I.” Vitamin MK-4 synthesis, simplified by omitting the polyisoprenoid-chain-elongation procedure, involved the same techniques, where the side chain building block consisted directly of E,E-farnesyl bromide. Interchangeable employment of geraniol and farnesol in the proceedings resulted in a variety of new building blocks, allowing for customisation of the side-chain length with ease. Geranylgeranyl and geranylfarnesyl intermediates were obtained, yielding vitamins MK-5 and MK-6, respectively. The purity assessment of the vitamins was based on the previously described method [18].

### 2.3. Anti-Inflammatory Effect of Vitamins K in RAW 264.7 Cells

To determine the anti-inflammatory effect of various forms of vitamins K, cells were seeded (5 × 10^5^ cells/well) in 24-well plates and incubated 48 h in presence or absence of vitamins K (0, 0.1, 1 and 10 μM, including 24 h pre-incubation with vitamins before pro-inflammatory stimulation with LPS (from Salmonella enterica serotype Abortusequi 1 ng/mL) adopting an experimental approach described previously [10]. Vitamins K at a concentration of 10 μM did not influence the viability of RAW264.7 cells as confirmed using the MTT test. The concentration of LPS (1 ng/mL) and time of pro-inflammatory stimulation (24-h) were chosen based on a preliminary experiment. All experiments were performed in dark conditions to protect the loss of physiological activity of the vitamins K analogues. The medium was changed every 24 h to ensure the presence of fresh vitamins K.

### 2.4. Measurements of Cytokines Production by RAW 264.7 Cells

The levels of TNFα and IL-6 in the cell culture supernatant were determined via the enzyme-linked immunosorbent assay (ELISA) with a commercially available kit (R&D system, Abingdon, UK) according to the manufacturer’s instructions.

### 2.5. Measurements of NO Production in RAW 264.7 Cells

The NO production was assessed based on the measurements of the concentration of nitrite (NO_2_^−^) and nitrate (NO_3_^−^) in cell supernatants by the ENO-20 NOx Analyzer (Amuza Inc., San Diego, CA, USA), as described previously [19]. In brief, the ENO-20 applies the liquid chromatography method with post-column derivatisation using a Griess reagent. In this method, nitrite and nitrate were separated on the NO-PAK column (4.6 × 50 mm, Amuza Inc., San Diego, CA, USA). Next, nitrate was reduced to nitrite by cadmium-cooper column (NO- RED, Amuza, Inc., San Diego, CA, USA), mixed with a Griess reagent and the absorbance of formed purple azo derivatives was measured at 540 nm.

### 2.6. UHPLC-MS/MS Measurements of Eicosanoid Production by RAW 264.7 Cells

The concentrations of PGE_2_, PGD_2_, PGF_2α_, 11β-PGF_2α_, 15-deoxy-PGJ_2_ and 8-iso-PGF_2α_ were measured using a UFLC Nexera liquid chromatograph system (Shimadzu, Kyoto, Japan) coupled to a QTrap 5500 triple quadrupole mass spectrometer (Sciex, Framingham, MA, USA). Briefly, samples were spiked with the internal standard (IS) mixture and extracted using ethyl acetate (EtOAc) acidified with acetic acid. After vigorous shaking, samples were centrifuged, and the upper organic layer was evaporated to dryness under a nitrogen stream (37 °C). The dry residue was reconstituted in nitrogen-purged ethanol (EtOH) and injected onto an Acquity UHPLC BEH C18 (3.0 × 100 mm, 1.7 µm, Waters, Milford) analytical column. Analytes were separated under the gradient elution mode. The MS detection of studied eicosanoid and their deuterated internal standards was carried out in negative ion electrospray ionisation by applying the multiple reaction monitoring mode (MRM); this method and the instrument operation parameters were described previously [20].

The quantification of studied eicosanoid was performed based on the calibration curves plotted for each eicosanoid as the relationship between the peak area ratios of analyte/IS to the nominal concentration of the analyte. Eicosanoid levels in cell medium samples were calculated based on the regression equations estimated for each analyte.

### 2.7. Measurements of Expression of NOS-2, COX-2 and MMPs in RAW 264.7 Cells

The expression of NOS-2, COX-2, MMP-2 and MMP-9 was assessed by the Western blot method. Cells were washed three times with Dulbecco’s phosphate-buffered saline (DPBS) (Gibco, Paisley, Scotland, UK) and lysed in a mammalian lysis buffer with protease and phosphatase inhibitors (Thermo Fisher Scientific, Waltham, MA, USA). Protein concentration was measured with the use of a BCA assay. The samples were reduced in a loading buffer and desaturated by heating at 95 °C for 5 min. An equal amount of protein from samples of 3 independent biological experiments was loaded and run on the gel and then transferred to a PVDF membrane, activated in methanol, blocked with 5% dry milk and incubated with the primary antibodies directed against the following antigens: COX-2 (16012; Cayman Chemicals, Ann Arbor, MI, USA), NOS-2 (PA3-030A; Thermo Fisher Scientific, Waltham, MA, USA), MMP-2 (ab19167; Abcam, Cambridge, UK) and MMP-9 (ab19016; Abcam, Cambridge, UK) and the appropriate horseradish peroxidase (HRP)-conjugated secondary antibodies (Santa Cruz Biotechnology, Texas, DA, USA). Equal protein loading was confirmed after transfer onto membranes, as measured by a stain-free technique provided by Bio-Rad [21]. Densitometric assessment of band intensity was performed using Image Lab software (Bio-Rad, Warsaw, Poland). The results are presented as the fold change of control corresponding to LPS-stimulated RAW 264.7 cells, without vitamins K. Total protein was used as a loading control.

### 2.8. Measurements of Mitochondrial Function in RAW 264.7 Cells

To assess effects of vitamins K on mitochondrial function, RAW 264.7 cells were seeded (6.5 × 10^3^ cells/well) into 96-well Seahorse plates and incubated 48 h with 1 μM of vitamin K_3_ or 10 μM of other vitamins K. The Seahorse XFe 96 Analyzer was used to measure the oxygen consumption rate (OCR; reflecting mitochondrial respiration) and extracellular acidification rate (ECAR; reflecting glycolysis). Measurements of OCR and ECAR were performed simultaneously according to protocols used previously [22]. Sequential injection of modulators of respiration that target components of the electron transport chain (ETC)—oligomycin (inhibitor of ATP synthase), FCCP (uncoupler), rotenone with antimycin A (respectively, inhibitors of complex I and complex III in the ETC)—enabled determination of the following parameters of mitochondrial function: basal respiration, proton leak, ATP-linked respiration, maximal respiration, reserve capacity and non-mitochondrial oxygen-consumption, as described previously [22].

### 2.9. Measurements of Endogenous Content of Vitamins K in RAW 264.7 Cells

RAW 264.7 cells were seeded (1 × 10^6^) in 24-well plates and incubated with exogenous vitamins K (K_1_, K_3_, MK-4, MK-5, MK-6 and MK-7) 24, 48 or 72 h. To assess the involvement of UBIAD-1 in endogenous vitamins K synthesis, the cells were incubated in the presence of atorvastatin (1 μM) shown to effectively inhibit UBIAD-1/MK-4 production, accordingly to literature data [23].

After 24, 48 or 72 h incubation, cells were washed with DPBS. Then, the cells were scraped off in 500 μL deionised distilled water and lysed by 10 min sonification. Cell lysates, as well as the post-culture medium, were kept at −80 °C until further analysis, i.e., LC-MS/MS quantification of vitamins K.

### 2.10. UHPLC-MS/MS Quantification of Vitamins K

The concentration of vitamins K (K_1_, MK-4 and MK-7) and CoQ10 was determined in the cell sample and post-culture medium using ultra pressure liquid chromatography coupled to tandem mass spectrometry with an atmospheric pressure chemical ionisation (UHPLC-APCI-MS/MS) technique, as described previously [24]. After the sample thawing, the solution of an internal standard containing K_1_-d_7_ (10 µL) was added, obtaining its final concentration of 100 ng/mL. Next, the ethanol (500 µL) was added after gentle mixing (10 min). The analytes were extracted using hexane (3 mL). After 10 min of vigorous shaking, the samples were centrifuged (4500× *g*, 10 min, 4 °C), and the upper organic layer was transferred to a fresh glass tube. The extraction step was repeated, and the whole collected organic layer was evaporated to dryness under a nitrogen stream (37 °C). The sample residue was reconstituted in 50 μL of 2-propanol, and 5 μL was injected into the UPLC-MS system. The chromatographic analysis was carried out using an Ultimate 3000 HPLC system (Dionex, Sunnyvale, CA, USA) equipped with a reversed-phase PFP analytical column (Kinetex 2.6 μm PFP, 100 Å, 100.0 × 3.3 mm; Phenomenex, Torrance, CA, USA). The mobile phase consisted of 0.1% formic acid in 2-propanol, and 0.1% formic acid in 5 mM ammonium formate was delivered in gradient elution mode. The signal from analytes and their internal standards was registered by applying a TSQ Quantum Ultra triple quadrupole mass spectrometer (Thermo Fisher Scientific, Waltham, MA, USA) in positive ionisation mode. The following most abundant ion transitions were monitored for quantification in selected reaction monitoring mode (SRM): K_1_: 451.4→187.2; K_1_-d_7_: 458.5→194.2; MK-4: 445.4→187.2; MK-4-d_7_: 452.4→194, MK-7: 649.6→187.2 and CoQ10: 863.6→197.1. The working parameters of the mass spectrometer were as follows: corona discharge needle voltage: 4 kV; vaporiser temperature: 325 °C; sheath gas pressure: 50 arb; ion sweep gas pressure: 10 arb; auxiliary gas pressure: 30 arb; capillary temperature: 325 °C; and collision pressure 1.5 mTorr, using argon as the collision gas.

The calibration curves were plotted as the relationship between the peak area ratios of the analyte/internal standard to the nominal concentration of the analyte. Vitamins K and CoQ10 levels in the studied samples were calculated based on the regression equations estimated for each analyte. The quantification of MK-4-d_7_ was performed based on the regression equations calculated for MK-4.

### 2.11. Statistical Analysis

Statistical analysis was performed using GraphPad Prism 9 (San Diego, CA, USA). Depending on data distribution (tested with the Shapiro–Wilk or D’Agostino–Pearson omnibus normality test), data were presented as mean ± SD or median ± IQR, respectively, of at least three biological experiments, with a minimum of three technical replicates without outliers. Only post hoc *p*-values < 0.05 were considered significant.

## 3. Results

### 3.1. Effects of Vitamins K on LPS-Induced NO Production and Induction of NOS-2 in RAW 264.7 Cells

**Figure 1:**
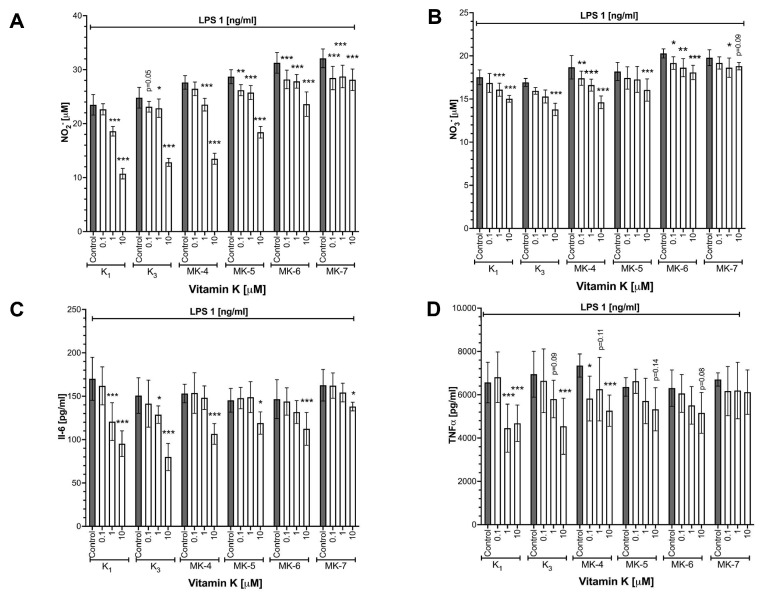
The inhibition of NO (NO_2_^−^, NO_3_^−^) and cytokine (IL-6, TNFα) production by various forms of vitamins K in RAW 264.7 cells stimulated with LPS. Nitrite (**A**) and nitrate (**B**) production was measured via the ENO-20-NOx Analyzer. The cytokine concentration, IL-6 (**C**) and TNFα (**D**), was measured using ELISA after 48 h incubation with various forms of 10 μM vitamins K in the presence of 1 ng/mL LPS. Data are shown as means ± SD from three independent experiments and technical replicates without outliers (*n* = 6–9). Statistical significance was evaluated by two-way ANOVA followed by Dunnett’s post hoc multiple comparisons using GraphPad Prism 9 software. The symbols *, ** and *** indicate statistical significance at *p* < 0.05, 0.01 and 0.001, respectively.

RAW 264.7 cells were stimulated with LPS (1 ng/mL, 24 h) in the presence or absence of various forms of vitamins K (K_1_, K_3_, MK-4_,_ MK-5, MK-6 and MK-7) at a concentration of 0, 0.1, 1 and 10 μM added 24 h before LPS stimulation. As shown in Figure 1, all forms of vitamins K inhibited nitrite (Figure 1A) and nitrate (Figure 1B) accumulation in a concentration-dependent manner, with the most pronounced effect at a concentration of 10 μM. The strongest inhibitory effect on NO_2_^−^ production was noted for K_1_ (54.4% inhibition), MK-4 (51.2% inhibition), K_3_ (48.3% inhibition) and MK-5 (35.9% inhibition) forms, as compared with MK-6 (24.5% inhibition) and MK-7 (12.4% inhibition), which displayed weaker effects. To confirm that vitamins K inhibit NO production by the regulation of NOS-2 expression, a Western blot analysis was performed (Figure S1A,E). The inhibition of an LPS-induced increase in NOS-2 expression was visible for all forms of vitamins K.

### 3.2. Effects of Vitamins K on LPS-Induced Cytokines Production in RAW 264.7 Cells

All forms of vitamins K (K_1_, K_3_, MK-4, MK-5, MK-6 and MK-7) reduced the LPS-induced production of IL-6 (Figure 1C) and TNFα (Figure 1D) in RAW 264.7 cells. The strongest inhibitory effect on cytokine production was observed for K_1_, K_3_ and MK-4, as compared with vitamins K forms with longer side chains, such as MK-6 and MK-7, which induced weaker effects.

### 3.3. Effects of Vitamins K on LPS-Induced Eicosanoid Production and Induction of COX-2 in RAW 264.7 Cells

**Figure 2:**
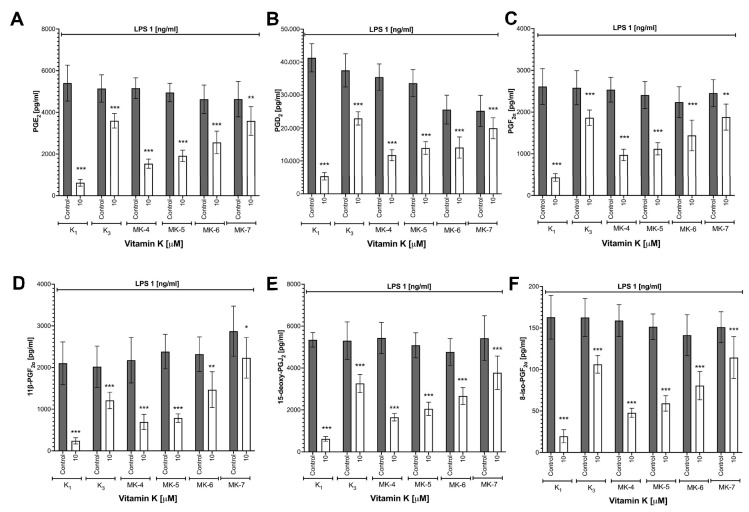
The inhibition of eicosanoid production by various forms of vitamins K in RAW 264.7 cells stimulated with LPS. PGE_2_ (**A**), PGD_2_ (**B**), PGF_2α_ (**C**), 11β-PGF_2α_ (**D**), 15-deoxy-PGJ_2_ (**E**) and 8-iso-PGF_2α_ (**F**) were measured in medium using the UHPLC-MS/MS technique in post-culture after 48 h incubation with various forms of 10 μM vitamins K in the presence of 1 ng/mL LPS (24 h). Data are shown as means ± SD from three independent experiments and technical replicates without outliers (*n* = 8–9). Statistical significance was evaluated by two-way ANOVA followed by Sidak’s post hoc multiple comparisons using GraphPad Prism 9 software. The symbols *, ** and *** indicate statistical significance at *p* < 0.05, 0.01 and 0.001, respectively.

As shown in Figure 2, all forms of vitamins K inhibited eicosanoid (PGE_2_, PGD_2_, PGF_2α_, 11β-PGF_2α_, 15-deoxy-PGJ_2_ and 8-iso-PGF_2α_) production in a concentration-dependent manner. The strongest inhibitory effect on eicosanoid production was observed for vitamin K_1_: PGE_2_ (88.5% inhibition), PGD_2_ (87.1% inhibition), PGF_2α_ (83.5% inhibition), 11β-PGF_2α_ (88.5% inhibition), 15-deoxy-PGJ_2_ (88.4% inhibition) and 8-iso-PGF_2α_ (88.1% inhibition); MK-7, in contrast, afforded weaker effects: PGE_2_ (22.6% inhibition), PGD_2_ (21.0% inhibition), PGF_2α_ (23.4% inhibition), 11β-PGF_2α_ (22.3% inhibition), 15-deoxy-PGJ_2_ (30.4% inhibition) and 8-iso-PGF_2α_ (24.2% inhibition). To confirm that vitamins K affected eicosanoid production by the regulation of COX-2 expression, a Western blot analysis was performed. All forms of vitamins K inhibited LPS-induced increase in COX-2 (Figure S1B) without an effect on COX-1 expression (data not shown).

### 3.4. Effects of Vitamins K on LPS-Induced MMP-2 and MMP-9 Expression in RAW 264.7 Cells

All examined form of vitamins K (K_1_, K_3_, MK-4, MK-5, MK-6 and MK-7) inhibited LPS-induced MMP-2 as well as MMP-9 protein expression (Figure S1C,D,G,H). However, the inhibition of the expression of MMP-2 protein seemed to be more pronounced than the inhibition of MMP-9 appeared.

### 3.5. Synthesis of Endogenous MK-4 from Exogenous Vitamins K

**Figure 3:**
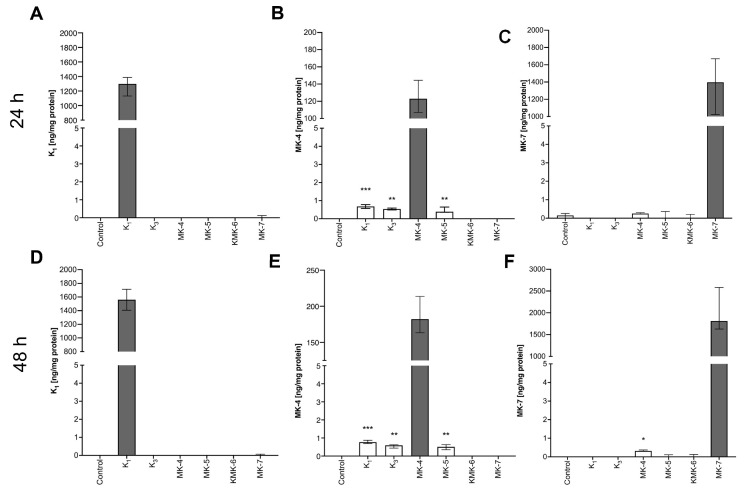
The intracellular concentration of vitamins K_1_, MK-4 and MK-7 in RAW 264.7 cells treated with 10 μM exogenous vitamins K (K_1_, K_3_, MK-4, MK-5, MK-6 and MK-7). To assess the interconversion of exogenous vitamins K to endogenous MK-4, RAW 264.7 cells were incubated 24 and 48 h with various forms of exogenous vitamins K. Then, the concentrations of K_1_ (**A**,**D**), MK-4 (**B**,**E**) and MK-7 (**C**,**F**) were measured in cell lysates using the UHPLC-MS/MS method. Data are shown as median and IQR of three independent experiments and technical replicates (*n* = 9). Statistical significance was evaluated by the Kruskal Wallis test followed by Dunn’s post hoc multiple comparisons using GraphPad Prism 9 software. The symbols *, ** and *** indicate statistical significance at *p* < 0.05, 0.01 and 0.001, respectively. The measured vitamins K, which was added exogenously to the medium, were excluded from the statistical analysis (indicated by the black in the graph).

Exogenous vitamins K_1_, K_3_ and MK-5, but not MK-6 or MK-7, resulted in an increase in endogenous MK-4 content in RAW 264.7 cells after 24 h (Figure 3B), 48 h (Figure 3E) and 72 h (data not shown) of incubation.

To confirm that endogenous MK-4 may be produced from exogenous K_1_ and K_3_, deuterium-labelled K_1_ (K_1_-d_7_) and K_3_ (K_3_-d_8_) vitamins were used. Twenty-four hours’ incubation of RAW 264.7 cells with K_1_-d_7_ and K_3_-d_8_ resulted in an increased intracellular concentration of vitamin MK-4-d_7_ analysed in cell lysate (Table 1).

### 3.6. Effects of Atorvastatin on Endogenous Production of MK-4 from Exogenous Vitamins K and on Anti-Inflammatory Effect of Vitamins K

**Figure 4:**
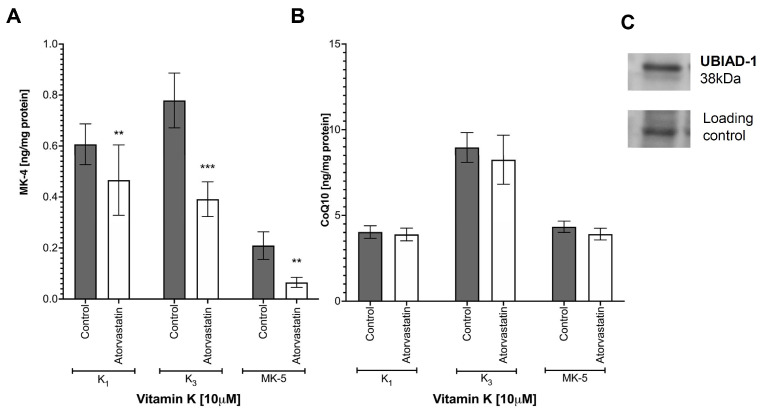
The inhibition of endogenous MK-4 synthesis by atorvastatin in RAW 264.7 cells. To measure MK-4 (**A**) and CoQ10 (**B**) levels, cells were incubated 48 h with 10 μM vitamins K_1_, K_3_ and MK-5 in the presence or the absence of 1 μM atorvastatin. Then, the concentration of endogenous MK-4 and CoQ10 were measured in cell lysates using UHPLC-MS/MS. Data are shown as means of three independent experiments and technical replicates (*n* = 9). Statistical significance was evaluated by two-way ANOVA followed by Sidak’s post hoc multiple comparisons using GraphPad Prism 9 software. The symbols ** and *** indicate statistical significance at *p* < 0.01 and 0.001, respectively. To confirm the presence of UBIAD-1 protein in RAW 264.7 cells, a Western blot was performed (**C**).

To test whether endogenous production of MK-4 from vitamin K_1_, K_3_ or MK-5 involves an UBIAD-1-dependent pathway, exogenous vitamin K_1,_ K_3_ or MK-5 (Figure 4A) were incubated in the absence or the presence of atorvastatin (1 μM). As shown in Figure 4 atorvastatin (1 μM) significantly decreased endogenous production of MK-4 from exogenous vitamins K_1_, K_3_ and MK-5. However, in the presence of atorvastatin, CoQ10 content in macrophages was not altered (Figure 4B). The presence of UBIAD-1 protein in the murine macrophages cell line, RAW 264.7, was confirmed by Western blot (Figure 4C).

**Figure 5:**
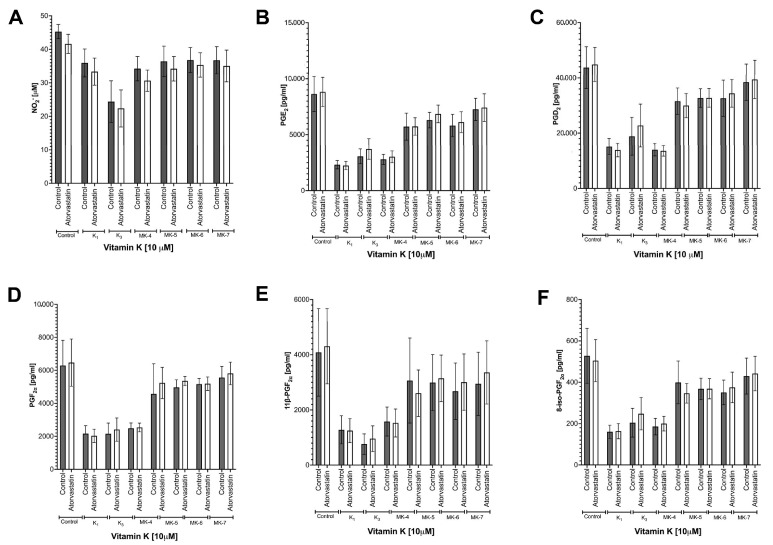
The lack of involvement of endogenous MK-4 in anti-inflammatory activity of exogenous vitamins K in RAW 264.7 cells stimulated with LPS. To determine the influence of vitamins K interconversion on anti-inflammatory activity of various forms of vitamins K, the production of endogenous MK-4 was blocked using 1 μM atorvastatin. Then, NO_2_^−^ (**A**) and eicosanoid (**B**–**F**) production were measured. Data are shown as means ± SD of three independent experiments with technical replicates excluding outliers (*n* = 6–9). Statistical significance was evaluated by two-way ANOVA followed by Sidak’s post hoc multiple comparisons using GraphPad Prism 9 software.

Despite the inhibitory effects of endogenous production of MK-4 by atorvastatin, the anti-inflammatory effect of exogenous K_1_, K_3_ and MK-5 remained unchanged by atorvastatin, as evidenced by the unmodified LPS-induced accumulation of nitrite and eicosanoid: PGE_2_, PGD_2_, PGF_2α_, 11β-PGF_2α_, 15-deoxy-PGJ_2_ and 8-isoPGF_2α_ (Figure 5).

### 3.7. Effects of Vitamins K on Mitochondrial Respiration

**Figure 6:**
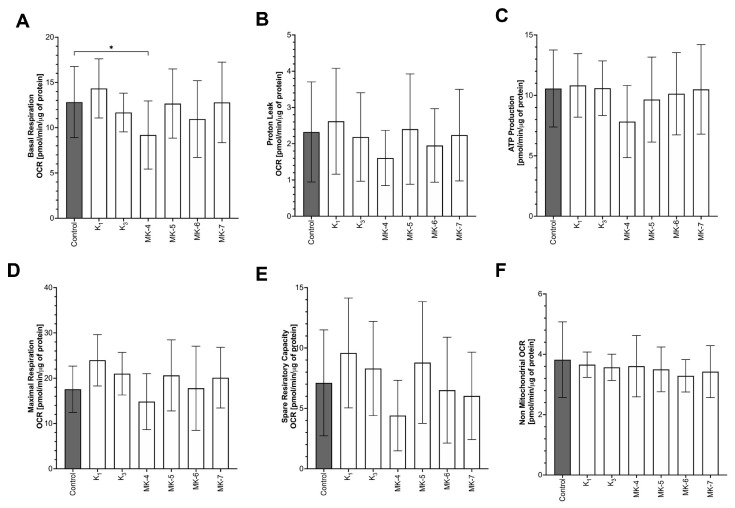
Effects of various forms of vitamins K on mitochondrial functions of RAW 264.7 cells. The mitochondrial function, in particular: basal respiration (**A**), proton leak (**B**), ATP production (**C**), maximal respiration (**D**), spare respiratory capacity (**E**) and non-mitochondrial oxygen-consumption (**F**) were analysed using the Seahorse method after 24 h incubation with various forms of vitamins K: K_1_, MK-4, MK-5, MK-6, MK-7 (10 μM) and K_3_ (1 μM). Data are shown as means ± SD of four independent experiments with technical replicates excluding outliers (*n* = 22–24). Statistical significance was evaluated by two-way ANOVA followed by Sidak’s post hoc multiple comparisons using GraphPad Prism 9 software. The symbol * indicates statistical significance at *p* < 0.05.

The influence of vitamins K on mitochondrial function was comprehensively analysed using the Seahorse method. None form of vitamins K (K_1_, K_3_, MK-4, MK-5, MK-6 or MK-7) improved mitochondrial function in non-stimulated macrophages, as evidenced by unchanged basal respiration, maximal respiration, ATP production, spare respiratory capacity, proton leak and non-mitochondrial oxygen consumption in the presence and absence of vitamins K (Figure 6).

## 4. Discussion

In the present work, we characterise the pharmacological effects of a wide range of forms of vitamins K (K_1_, K_3_, MK-4, MK-5, MK-6 and MK-7) added exogenously to LPS-stimulated RAW 264.7 cells and demonstrated their anti-inflammatory actions, which included the following: (1) the downregulation of the induction of NOS-2 and NO production, (2) the downregulation of COX-2 induction and various eicosanoid production (PGE_2_, PGD_2_, PGF_2α_, 11β-PGF_2α_, 15-deoxy-PGJ_2_ and 8-isoPGF_2α_), (3) the inhibition of cytokine production (IL-6 and TNFα) and (4) the inhibition of MMP-2 and MMP-9 expression. Interestingly, the vitamins K forms with shorter isoprenoid chains, such as K_1_, K_3_, MK-4 and MK-5, displayed stronger anti-inflammatory activity than the forms with longer isoprenoid chains (MK-6 and MK-7). The nexus of our results is our demonstration, which is to our knowledge for the first time, that endogenous MK-4 synthesis is activated in RAW 264.7 cells when some forms of exogenous vitamins K, including K_1_, K_3_ and MK-5 as substrates, are available. Finally, our results showed that endogenous MK-4 synthesis was inhibited by atorvastatin, while the anti-inflammatory effects of exogenous vitamins K were not. Altogether, our results suggest that macrophages display an active synthesis of endogenous MK-4 from some forms of exogenous vitamins K that could be inhibited by statin, but anti-inflammatory effects of exogenous vitamins K could not be attributed to endogenous MK-4, but could be ascribed to the inhibition of NFκB-dependent pathway [12].

Our work extends previous reports on the anti-inflammatory effects of vitamins K. Previously, the anti-inflammatory effects of vitamin K_1_ [13], MK-4 [9] MK-3 and MK-7 [10] on cytokine production (e.g., IL-1, IL-6 and TNFα) were demonstrated, but prior research did not investigate any such effects on other inflammatory pathways, such as COX-2, NOS-2 or MMP. This study also incorporated a broader spectrum of forms of vitamins K than previous studies; the anti-inflammatory effects of K_1_ [13], K_3_ [25], MK-4 [9] and MK-7 [10], but not MK-5 and MK-6 were previously characterised. We confirmed anti-inflammatory effects in the murine macrophages cell line, RAW 264.7, for all forms of vitamins K (K_1_, K_3_, MK-4, MK-5, MK-6 and MK-7), but striking differences in their potency were observed. Specifically, there were stronger anti-inflammatory effects of K_3_ and forms of vitamins K with shorter isoprenoid chains (K_1_, MK-4 and MK-5) than among MK-6 and MK-7, which contain longer isoprenoid chains. This pattern diverged, for example, from endothelial cells, where MK-7 displayed more potent effects than MK-4 [26], as well as in the case of in vivo model, where MK-7 was shown to be more efficient to achieve carboxylation of extrahepatic (e.g., osteocalcin) and hepatic (e.g., prothrombin) proteins [27]. The latter could be due to the advantageous pharmacokinetic profile of MK-7, but the order of potency of binding of K_1_, MK-4, MK-7 and K_3_ to VCORC1 and the efficacy of various forms of vitamins K to serve as substrates for post-translational γ-carboxylation pointed to the weakest effects for K_3_ [28]; in contrast, in our experiments, K_3_ was among the more potent forms of vitamins K in inducing anti-inflammatory effects in RAW 264.7 cells.

To understand these findings more effectively, we analysed in detail the intracellular metabolism of vitamins K in macrophages by using deuterium-labelled K_1_ (K_1_-d_7_) and K_3_ (K_3_-d_8_) vitamins and our recently established UHPLC-MS/MS-based method of vitamins K quantification [24]. Using this approach, we provided unequivocal evidence that the macrophages produced endogenous vitamin MK-4 from exogenous vitamin K_1_, K_2_ or K_3_.

The production of endogenous MK-4 from exogenous precursors has been described in intestinal bacteria, the kidneys, the brain, the intestines and the endothelium [29,30,31,32] but not in macrophages.

It has been previously reported that intestinal bacteria or epithelial cells in the intestines have specific enzymes producing menadione from dietary exogenous vitamin K_1_ [32]. However, prenylation of menadione moiety could be executed in a number of cells via UBIAD-1, the enzyme involved in the intracellular de novo cholesterol synthesis [33]. In the present study, we observed the unique pattern of interconversion of exogenous vitamins K in RAW 264.7 cells to endogenous MK-4 synthesis. Exogenous K_1_, K_3_ and MK-5, but not MK-6 and MK-7, were used as exogenous substrates for endogenous MK-4 synthesis in macrophages, suggesting that shorter, unsaturated isoprenoid chains of vitamin K_2_; monounsaturated phytol sidechains (vitamin K_1_); or ready-to-use menadione (K_3_) [34] represent preferable substrates for endogenous MK-4 synthesis as compared with the longer unsaturated isoprenoid chain, which is harder to cleave [35]. Surprisingly, the inhibitory effect of atorvastatin was considerably weaker for endogenous production of MK-4 from vitamin K_1_ as compared with the effects of atorvastatin on the endogenous production of MK-4 from K_3_. Again, the explanation of this phenomenon may be the fact that menadione is ready to use substrate to prenylation, while in the case of K_1_, firstly, the isoprenoid side chain has to be cleaved. It could well be that the longer time of incubation with atorvastatin could result in a stronger inhibition of the conversion of K_1_ into MK-4. Additionally, we noted MK-7 synthesis after exogenous MK4 administration, but to explain this phenomenon, further research is needed.

Our results clearly indicate that macrophages have full machinery for the deprenylation of exogenous vitamins and, subsequently, prenylation of menadione for the synthesis of endogenous vitamin MK-4. In contrast to endothelial cells [34], coenzyme Q10 (CoQ10) content in macrophages was not inhibited by atorvastatin, but in our hands, the endogenous CoQ10 content was several folds higher than MK-4 content and higher than CoQ10 content in endothelial cells.

To summarise, macrophages extend the list of cells, including the bacteria, intestinal epithelium, kidneys, the brain, and the endothelium [30,31,32,36,37], that are able to synthesise MK-4. Macrophages displayed the ability to synthesise endogenous vitamin MK-4 from various exogenous sources of vitamins K that could originate from the diet or bacteria. Whether this pattern represents a cell-specific feature is not known; previous work reporting turnover into endogenous MK-4 in various cells examined only vitamin K_1_, K_3_ or MK-7, not the whole spectrum of exogenous forms of vitamins K, as used in the present study [32,38,39,40]. Generally, it is believed that MK-4 is produced in the body after K_1_ or MK-7 administration in vivo [41]. At the same time, it is accepted that menaquinone and phylloquinone have different functions: vitamin K_2_ mainly exerts extrahepatic activity, including vasoprotective action, while vitamin K_1_ primarily engenders hepatic activity and regulates coagulation. In contrast to this notion, our data demonstrate that various forms of vitamins K, including exogenous K_1_ and MK-4, exhibit anti-inflammatory activity.

In the present work, we did not identify the enzyme or enzymes involved in the cleavage of side chains of exogenous vitamins K, but we indicated that the prenylation activity was related to UBIAD-1 activity and was inhibited by statins. In fact, atorvastatin inhibited endogenous MK-4 production, but without an impact on the anti-inflammatory effects of vitamins K. Consequently, our results demonstrated that the anti-inflammatory effects of exogenous vitamins K in murine macrophages could not be explained by endogenous MK-4 synthesis despite active MK-4 synthesis in the cells. On the other hand, it should be noted that treatment with exogenous vitamins at micromolar concentration resulted in the nanomolar concentration of endogenous MK-4. Therefore, we are not able to exclude that endogenous MK-4—if achieving micromolar concentration—would inhibit the inflammatory response.

Obviously, endogenous MK-4 might be involved in the regulation of macrophage function, for example, via the regulation of senescence-related genes, regulation of differentiation, autophagy and apoptosis [42,43] or the regulation of proliferation [44,45]. Further studies are needed to define the functional role of endogenous MK-4 in macrophages.

Previous studies indicate the role of reduced forms of vitamins K in vitamins K-dependent carboxylation in various processes, and the number of VKDPs has been identified [8,46]. Although we confirmed the presence of GGCX protein in RAW 264.7 cells via Western blot, the VCOR blockade using two different antagonists of vitamins K (acenocoumarol and warfarin) had no impact on the anti-inflammatory effects of vitamin K_1_, K_3_ and MK-4, as assessed on the basis of NO_2_^−^ production (data now shown) thereby confirming the previous reports indicating that regulation of inflammation by vitamins K was independent of VKD carboxylation in macrophage-like cells [13,14]. Indeed, in THP-1 cells stimulated with LPS, the inhibition of VKOR by warfarin had no impact on the inhibitory activity of vitamin MK-4 on IL-6 mRNA expression [13]. Furthermore, the anti-inflammatory activity of the Gla-rich protein was observed for carboxylated as well as non-carboxylated forms of protein despite the changes of expression of GGCX protein in response to vitamins K treatment [11,14]. Altogether, the presence and changes of GGCX expression in response to vitamins K suggest that VKD carboxylation can be somehow involved in the regulation of macrophage function but that the anti-inflammatory effects of various forms of vitamins K reported here were independent of γ-carboxylation.

Finally, in the present work, we also excluded the mitochondrial-targeted mechanisms of the anti-inflammatory action of vitamins K. Even though mitochondria play an important role in inflammation [47] and vitamin K_2_ can act as a membrane-bound electron carrier in bacteria [16,17], our results demonstrated that vitamins K had no impact on mitochondrial function in the murine macrophages cell line, RAW 264.7, as evidenced by unchanged parameters of mitochondrial respiration such as basal respiration, ATP-linked respiration, maximal respiration and reserve capacity. It is likely that we were unable to observe the effects of vitamins K on mitochondrial function because of a large reserve of ubiquinone in RAW 264.7 cells. Indeed, the concentration of CoQ10 was approximately four times higher than the concentration of endogenous MK-4 in macrophages, as measured here by UHPLC-MS/MS. In fibroblasts, even in the state of CoQ10 deficiency, vitamin K_2_ did not act as an electron carrier [17].

Furthermore, the role of vitamins K as a ligand of nuclear steroid receptor (SXR) seems an unlikely mechanism of anti-inflammatory effects of vitamins K. Although previous studies reported that vitamin K_1_ or K_2_ [48,49,50,51] were ligands of steroid and xenobiotic receptor (SXR), the vitamin K_1_ was a weaker ligand of SXR than MK-4, as compared with the more potent or similar anti-inflammatory activity of vitamin K_1_ vs. MK-4 reported here.

Previous work demonstrated that K_3_ and their analogues inhibited NFκB activation and the phosphorylation of IKKα/β in macrophages stimulated with LPS [13]. Accordingly, the pattern of the anti-inflammatory profile of vitamins K described in the present work, which involves effects on NOS-2, COX-2, cytokines and MMPs known to be regulated by NFκB [52,53,54], suggest that the anti-inflammatory activity of vitamins K was mediated by menadione, the intermediate product of the endogenous conversion of vitamins K in macrophages. This notion is in line with the stronger anti-inflammatory potential of the shorter forms of vitamins to convert more easily into menadione [55].

In fact, our results confirmed the inhibitory function of vitamins K on NFκB nuclear translocation in LPS-stimulated RAW 264.7 cells (Figure S2). Thus, the anti-inflammatory effects of vitamins K could be ascribed to the regulation of the NFκB pathway [12,13]. However, the details of this mechanisms need to be further explored [56,57,58].

The anti-inflammatory activity of vitamins K in macrophages elaborated upon here and reported previously [10,13] represents an important asset of vitamins K action that may have a therapeutic significance in various inflammatory conditions [5,9,24,59,60,61,62]. In fact, high vitamin K_1_ status was associated with lower concentrations of pro-inflammatory markers in post-menopausal women [9]. In contrast, statins blocking endogenous MK-4 synthesis in macrophages, and presumably in many other cells, may have detrimental effects. Indeed, possible side effects of statins, which include myalgia [63], neuropathy [64] or diabetes [65], may be related to the inhibition of endogenous synthesis of MK-4. To conclude, despite the vast literature on vitamins K pharmacology, in the present work, we provided a novel insight into the profile of anti-inflammatory action of exogenous vitamins K. We demonstrated that anti-inflammatory effects of vitamins K_1_, K_2_ and K_3_ in LPS-induced macrophages encompass a wide spectrum of activities, including the inhibition of NOS-2/NO, COX-2/eicosanoid, cytokine and MMP-dependent pathways. Furthermore, our study provided evidence, to our knowledge for the first time, that besides the intestinal bacteria, kidneys, brain, intestines and the endothelium also macrophages are able to synthesise endogenous MK-4 vitamin. Finally, our results uncovered that various forms of exogenous vitamins K (K_1_, K_3_ and MK-5 but not MK-6, MK-7) provide a substrate for endogenous MK-4 synthesis in macrophages. However, the anti-inflammatory effects of various forms of vitamins K were independent of the UBIAD-1-dependent synthesis of endogenous MK-4 but ascribed to the inhibition of the NFκB pathway as suggested also previously [12,13]. The role of endogenous MK-4 in the regulation of the function of macrophages remains to be established.

## Figures and Tables

**Table 1:**
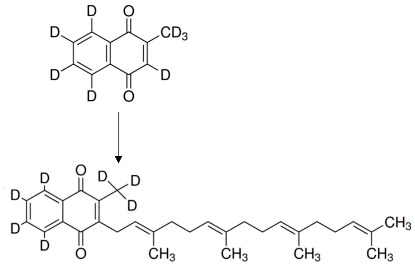
A comparison of endogenous production of MK-4/MK4-d_7_ in response to K_1_/K_1_-d_7_ or K_3_/K_3_-d_8_ treatment. RAW 264.7 cells were incubated 48 h with vitamin K_1_/K_1_-d_7_ or K_3_/K_3_- d_8_. Then, the concentration of MK-4/MK-4-d_7_ was measured in cell lysates using the UHPLC-MS/MS technique. Data are shown as median ± SD of three independent experiments with technical replicates (*n* = 9).

Exogenous Vitamins K	Endogenous MK-4 [ng/mg Protein]	Endogenous MK-4-d_7_ [ng/mg Protein]
K_1_	0.78 ± 0.13	-
K_1_-d_7_	-	0.65 ± 0.11
K_3_	0.53 ± 0.13	-
K_3_-d_8_	-	1.14 ± 0.33

## Data Availability

The data presented in this study are available on reasonable request from the corresponding author.

## Supplementary Materials

The following are available online at https://www.mdpi.com/article/10.3390/cells10071571/s1. Figure S1 Inhibition of NOS-2, COX-2, MMP-2 and MMP-9 expression by various forms of vitamins K in RAW 264.7 cells stimulated with LPS. Figure S2 Role of NFκB in the anti-inflammatory action of Vitamins K.
