# Supplementary material for: Exogenous Vitamins K Exert Anti-Inflammatory Effects Dissociated from Their Role as Substrates for Synthesis of Endogenous MK-4 in Murine Macrophages Cell Line
Source: Cells. 2021 Jun 22;10(7):1571. doi: 10.3390/cells10071571 (PMC8303864; doi:10.3390/cells10071571)
Supplement: Supplementary file 1 [file cells-10-01571-s001.zip › cells-1262335-supplementary.pdf]

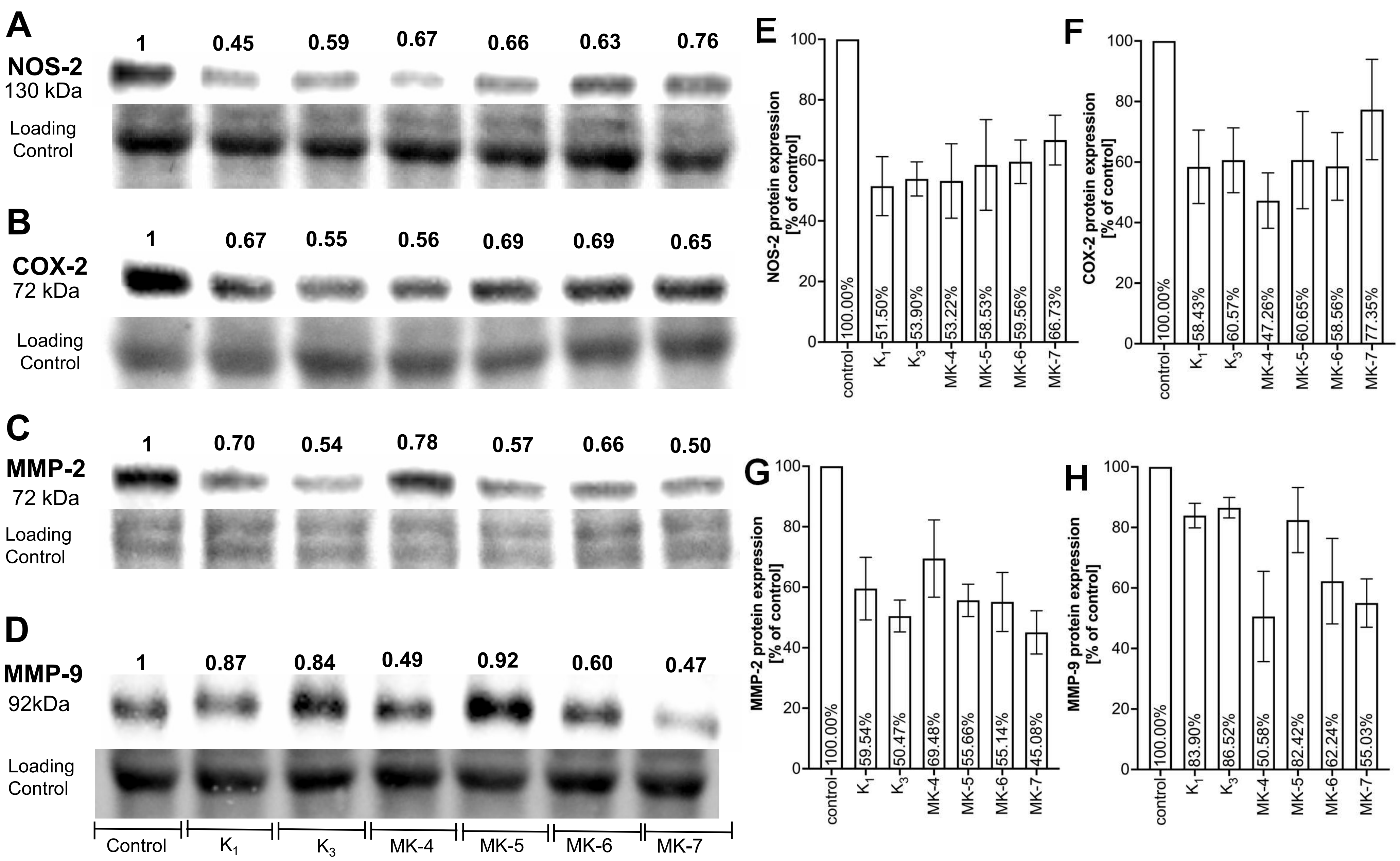

Figure. S1 Inhibition of NOS-2 , COX-2, MMP-2 and MMP-9 expression by various forms of vitamin K in RAW 264.7 cells stimulated with LPS. Western blot images from representative experiments present effect of vitamin K analogues on NOS-2 (A), COX-2 (B), MMP-2 (C) and MMP-9 (D) expression after 48 h incubation with various forms of 10 μM vitamin K in presence of 1ng/ml LPS as a fold change vs control sample (without vitamin K). Total protein after transfer was used as loading control using stain free technique (BioRad). Charts are shown as means of NOS-2 (E), COX-2 (F), MMP-2 (G) and MMP-9 (H) expression ± SD of three independent experiments. Percentage value are shown protein expression in response to vitamins K treatment (10 μM) as a fold change ± SD vs control sample (without vitamin K).

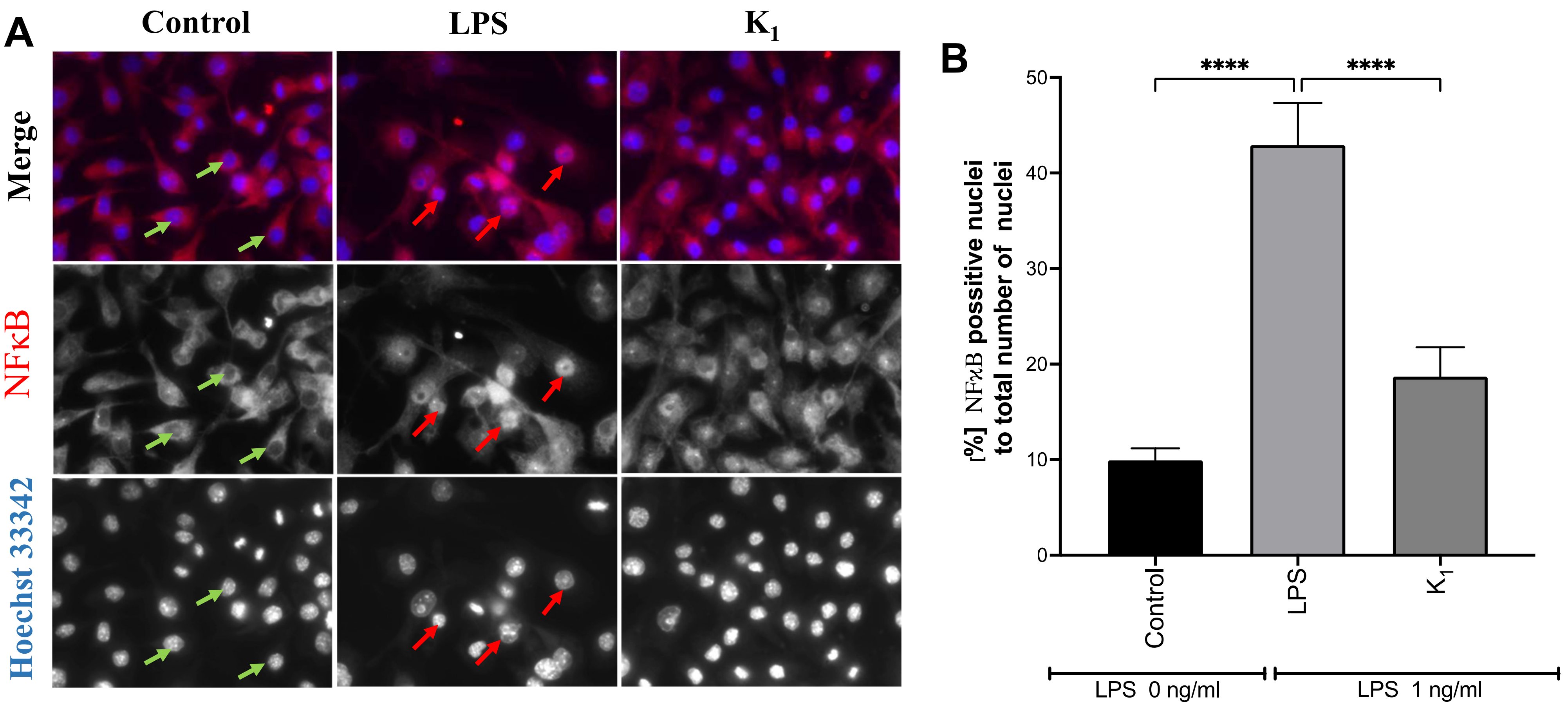

Figure. S2 Role of NFκB in the anti-inflammatory action of Vitamin K.. The importance of nuclear translocation NFκB (A,B) was assessed in RAW 264.7 murine macrophages stimulated with LPS. The changes of nuclear translocation of NFκB in response to vitamin K treatment (K_1) was demonstrated on representative pictures (A). The examples of NFκB immuno-negative and immuno-positive nuclei were marked with a green or red arrow respectively. Moreover the percentage of NFκB positive nuclei to all nuclei for each picture was shown in the graph as mean ± SEM of three independent experiments and three technical replicates and three pictures from each well, (n=9) (see more in **Materials and Methods** 2.6.) Statistical significance was evaluated by Two-way ANOVA followed by Dunnett's post-hoc multiple comparisons using GraphPad Prism 9 software. The symbol **** indicate statistical significance at $p < 0.0001$.
